# Infrared thermometry study of nanofluid pool boiling phenomena

**DOI:** 10.1186/1556-276X-6-232

**Published:** 2011-03-16

**Authors:** Craig Gerardi, Jacopo Buongiorno, Lin-wen Hu, Thomas McKrell

**Affiliations:** 1 Department of Nuclear Science and Engineering, Massachusetts Institute of Technology, 77 Massachusetts Ave., Cambridge, MA 02139 USA; 2 Nuclear Reactor Laboratory, Massachusetts Institute of Technology, 77 Massachusetts Ave., Cambridge, MA 02139 USA; 3 Argonne National Laboratory, Nuclear Engineering Division, 9700 S. Cass Ave., Argonne, IL 60439 USA

## Abstract

Infrared thermometry was used to obtain first-of-a-kind, time- and space-resolved data for pool boiling phenomena in water-based nanofluids with diamond and silica nanoparticles at low concentration (<0.1 vol.%). In addition to macroscopic parameters like the average heat transfer coefficient and critical heat flux [CHF] value, more fundamental parameters such as the bubble departure diameter and frequency, growth and wait times, and nucleation site density [NSD] were directly measured for a thin, resistively heated, indium-tin-oxide surface deposited onto a sapphire substrate. Consistent with other nanofluid studies, the nanoparticles caused deterioration in the nucleate boiling heat transfer (by as much as 50%) and an increase in the CHF (by as much as 100%). The bubble departure frequency and NSD were found to be lower in nanofluids compared with water for the same wall superheat. Furthermore, it was found that a porous layer of nanoparticles built up on the heater surface during nucleate boiling, which improved surface wettability compared with the water-boiled surfaces. Using the prevalent nucleate boiling models, it was possible to correlate this improved surface wettability to the experimentally observed reductions in the bubble departure frequency, NSD, and ultimately to the deterioration in the nucleate boiling heat transfer and the CHF enhancement.

## Introduction

Numerous studies have recently been produced on the heat transfer properties of common fluids whose properties have been modified through the addition of solid nanoparticles. The resulting colloidal suspensions are known in the literature as nanofluids (e.g., [1]). Previous studies of nanofluid pool boiling [2-14] have shown both a significant critical heat flux [CHF] enhancement (up to 200%) and an alteration of the nucleate boiling heat transfer coefficient (sometimes an enhancement, sometimes a deterioration). These studies found these phenomena to occur at low particle concentration (typically <1% by volume) and that the nanoparticles form a porous layer on the surface during nucleate boiling.

A recent review of the work done on nucleate pool boiling in nanofluids can be found in Das et al. [15]. Conflicting experimental results from researchers reporting heat transfer enhancement, deterioration, and no effect make it impossible to state a specific trend. However, it seems that the particle concentration and size have a significant impact on the reported results. Das et al. [15] reported that nanofluids that exhibit nucleate boiling heat transfer coefficient deterioration typically have high particle concentrations (4% to 16% by weight), while enhancement is typically found at relatively low particle concentrations (<1.25% by weight). Kim et al. [14], however, reported a significant decrease in heat transfer coefficients at low (<0.1% by volume) particle concentrations of alumina, silica, and zirconia nanofluids. Additionally, Kim et al. [16] demonstrated that the relative surface wettability of the deposited nanoparticles compared with a clean surface significantly affected the boiling performance. Narayan et al. [17] found both deterioration and enhancement of the heat transfer coefficient at relatively low alumina particle concentrations (0.5 to 2 wt.%) in water. They explained that this apparent conflict could be resolved when the ratio of the average surface roughness to the average particle diameter was accounted for. When this parameter was near unity, they found that boiling deterioration was the most dramatic, which they theorized was caused by the nanoparticles plugging the nucleation sites, inhibiting heat transfer.

While there are conflicting results in the literature for the modification of the nucleate boiling heat transfer coefficient, there is no dispute that CHF is enhanced due to nanofluid boiling [2-14]. The magnitude of this enhancement is highly variable, but all known works of research report an increase in CHF with nanofluid boiling. Most researchers used water as a base fluid, and nearly all researchers used low concentrations of nanoparticles (<1 vol.%) where the thermophysical properties of the base fluid are unaffected. The range of CHF enhancement reported by these researchers is from as little as 25% to as much as 200% over CHF for the base fluid. This is a very significant finding since such a substantial enhancement in the upper limit of nucleate boiling is found with little or no change in the thermophysical fluid properties. The most widely accepted mechanism for CHF enhancement in nanofluids is due to the enhanced wettability of the particle layer over the clean surface, as first proposed by Kim et al. [18]. Capillary wicking in porous structures has also been shown [19-21] to increase CHF for increased capillary length at fixed surface contact angles.

While all the aforementioned mechanisms and effects have been proposed and qualitatively studied to some extent in the literature, there is a lack of 'hard' experimental information on how these effects would influence the parameters that ultimately govern nucleate boiling, e.g., bubble departure diameter and frequency, wait time, and nucleation site density. Part of the problem is that such parameters, while recognized as important, are extremely difficult to measure. In this paper, we report and compare directly measured data for bubble departure diameter and frequency, growth and wait times, and nucleation site density for pure water and two water-based nanofluids, obtained using a state-of-the-art facility based on infrared thermometry [22,23]. The data are then analyzed to elucidate the mechanisms by which the nucleate boiling heat transfer coefficient and CHF are affected by the presence of the nanoparticles.

## Experimental

### Nanofluid preparation and characterization

Two nanoparticle materials, i.e., silica (SiO_2_) and diamond (C), were selected for these experiments primarily due to their high chemical and colloidal stability. Both nanoparticle types have also previously [16,24] been shown to have a positive influence on boiling phenomena at the concentrations used in this work. Water-based nanofluids of these nanoparticles were purchased as Ludox TMA from Sigma-Aldrich (silica; St. Louis, MO, USA) and Plasma-Chem GmbH (diamond; Berlin, Germany). The delivered concentrations of the silica and diamond nanoparticles were 34% and 4% by weight, respectively. The as-purchased nanofluids were then diluted with deionized water to the low concentrations of interest in these experiments, i.e., 0.1% by volume for silica and 0.01% by volume for diamond. The mean effective diameter of the nanoparticles in the dilute nanofluids was measured with the dynamic light scattering technique and was approximately 34 ± 10 nm (measured range from 16 to 50 nm) [25] for the silica nanofluid and 173 ± 10 nm (measured range from 90 to 377 nm) [26] for the diamond nanofluid. No surfactant was used to stabilize either nanofluid. Scanning electron microscope [SEM] pictures of the dried silica particles showed them to be very spherical [26]. Various properties relevant to two-phase heat transfer were also measured. The surface tension, thermal conductivity, and viscosity of the nanofluids were measured [26,27] by means of a tensiometer, a thermal conductivity probe, and a capillary viscometer, respectively. These properties were found to differ negligibly from those of pure water, i.e., within ± 4%. At the low concentrations of interest here, the fluid density and heat of vaporization can also be considered unaltered. The temperature dependence of viscosity and thermal conductivity for low nanoparticle loadings in water were measured by Williams et al. [28] and found to be the same as that of water. In summary, the transport and thermodynamic properties of the dilute nanofluids used in these experiments are very similar to those of pure water; thus, the thermo-physical properties of nanofluids are not expected to be responsible for any change in the heat transfer coefficient or critical heat flux.

### Boiling apparatus

**Figure 1:**
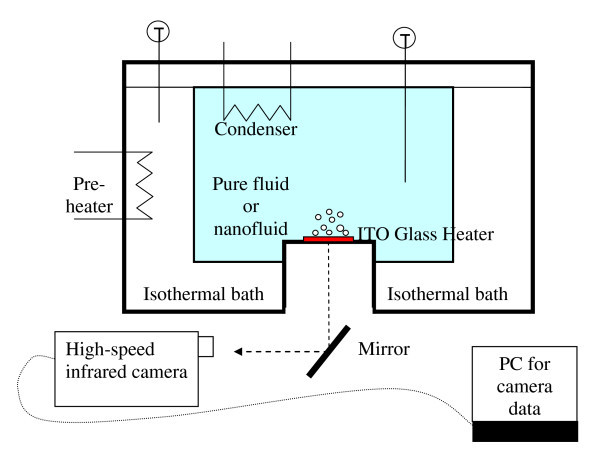
**MIT pool boiling facility with infrared thermometry**.

The experiments were conducted at saturation at atmospheric pressure in the facility shown in Figure 1. A 0.7-μm-thick film made of indium-tin-oxide [ITO] was resistively heated. Boiling occurred on the upward facing side of this film which had an exposed area of 30 × 10 mm^2^. The ITO was vacuum-deposited onto a 0.4-mm-thick sapphire substrate and connected to a direct current power supply to control the heat flux at the surface. The cell accommodating the test fluid was sealed, included a condenser, and was surrounded by a constant-temperature water bath to maintain a constant test fluid temperature by minimizing heat losses to the ambient.

Acquisition of the temperature distribution on the heater surface was accomplished using an infrared [IR] high-speed camera, SC 6000 from FLIR Systems, Inc. (N. Billerica, MA, USA). The use of an IR camera to investigate boiling heat transfer was pioneered by Theofanous et al. [29]. As configured in this study, the IR camera had a spatial resolution of 100 μm, which is more than sufficient to capture the temperature distribution about individual nucleation sites since the typical bubble diameter is on the order of 1,000 μm. The capture frame rate was 500 Hz. The raw data obtained for each heat flux are in the form of hundreds of frames, each representing a two-dimensional infrared intensity distribution on the heater surface (see [22]). The conversion from IR intensity to temperature is done via a calibration curve completed prior to each experiment by placing a thermocouple with an accuracy of approximately 2% (or 2°C) on the ITO surface while simultaneously capturing IR images. The IR camera has a sensitivity of 0.02°C.

While the sapphire substrate is transparent (>85%) to IR light, the ITO has the advantageous property of being opaque in the IR range as this ensures that all temperature measurements are made on the back (bottom) of the ITO substrate. The thinness of the ITO heater guarantees that the IR camera reading from its bottom was an accurate representation of the actual temperature on the top (wet side) of the heater surface. Thus, neither the temperature of the fluid nor the integral temperature through the substrate thickness was measured. This made thermal analysis of the heater and corresponding temperature measurements straightforward. Use of the IR camera (vs. the more traditional approach based on thermocouples embedded at discrete positions in the heater) enables mapping of the complete two-dimensional time-dependent temperature distribution on the heater surface. Heat loss from the heater bottom via air natural convection was calculated to be negligible (<1%).

During each experiment, the heat flux was increased in discrete steps (25 to 50 kW/m^2^) up to the CHF. At each intermediate step, the temperature map was recorded for 2.0 s. Since the typical timescale for a bubble nucleation cycle is tens of milliseconds, 2.0 s is sufficient to obtain good data statistics. Near the critical heat flux, the heat flux was increased in smaller increments (10 to 25 kW/m^2^) to ensure higher accuracy in capturing the CHF event.

A detailed discussion of the experimental procedure, data reduction procedure, and measurement uncertainty is available in a previously published study by the same authors on pool boiling heat transfer in water [22,23].

### Experimental results

**Figure 2:**
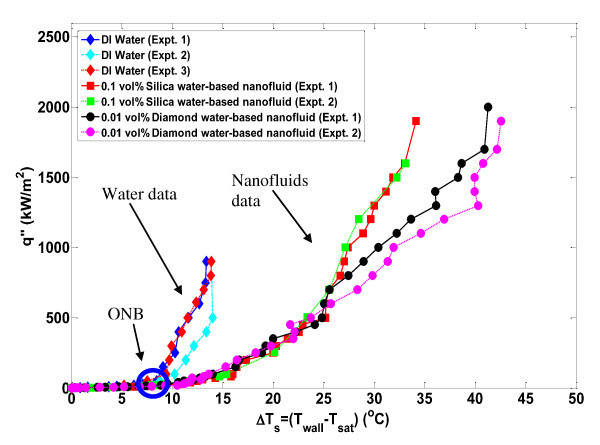
**Pool boiling curve for DI water and nanofluids tests systematically discussed in this work**. Approximate uncertainty in measurement of *q*" and Δ*T*s are both 2%. The ONB is at approximately the same superheat (~7°C) for all experiments (i.e., water and nanofluid ONB is very similar). ONB, onset of nucleate boiling.

**Figure 3:**
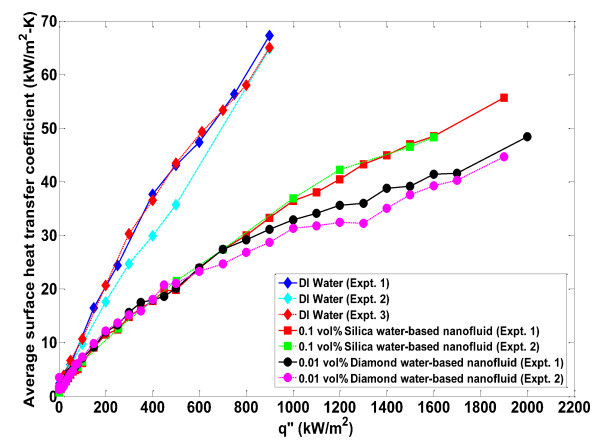
**Average wall heat transfer coefficient as a function of applied heat flux**. Uncertainty in heat transfer coefficient is ± 3%.

The nucleate boiling and critical heat flux characteristics of deionized water and water-based nanofluids were studied with infrared thermometry. Pool boiling curves (shown in Figure 2) were generated for the seven (three pure water and four nanofluid) experiments that are discussed in this paper by taking the time average (over 2.0 s) and space average (of a 5 × 5-mm^2 ^area in the center of the heater) of the IR-measured temperature distribution at a given heat flux. Several generalized conclusions can be immediately inferred by inspecting this figure. First, the effective nucleate boiling heat transfer coefficient for all nanofluids is lower (i.e., deteriorated) compared with the water experiments since the boiling curves are shifted significantly to the right. This reduction is further highlighted in Figure 3; here, the heat transfer coefficient is calculated from knowledge of the heat flux, the average measured surface temperature, and the bulk fluid temperature (which is the saturation temperature for these experiments)(1)

The reduction in nucleate heat transfer coefficient in nanofluids is as much as 50% for a given wall superheat. The second conclusion that can be made is that the value of critical heat flux in nanofluids was significantly higher (~100%) than the average water value. The critical heat flux values for all experiments run in this series are displayed in Table 1, including those where the boiling curve was not evaluated for plotting in Figure 2. The uncertainty in the CHF values was estimated to be ± 10%, which can primarily be attributed to the possibility that CHF could occur between discrete heat flux steps which were always <10% of the total heat flux near CHF.

**Table 1:** Summary of CHF results (uncertainty ± 10%)

Test fluid	**Expt. no**.	**Critical heat flux value (kW/m**^**2**^**)**	**Average critical heat flux value for test fluid (kW/m**^**2**^**)**
DI water	1	900	976
	2	1,080	
	3	900	
	4	1,000	
	5	1,000	

Nanofluid-Silica (0.1 vol.%) in water	1	1,800	1,767
	2	1,900	
	3	1,600	

Nanofluid-Diamond (0.01 vol.%) in water	1	2,000	1,950
	2	1,900	

Values for all experimental runs shown here including those where a boiling curve was not generated for Figure 2

**Figure 4:**
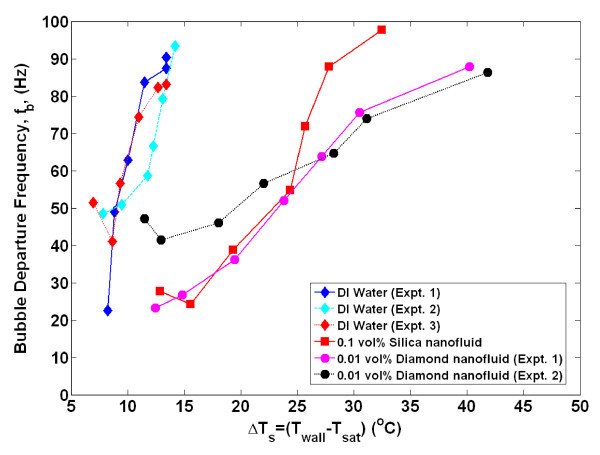
**Average bubble departure frequency as measured by infrared thermometry**. Uncertainty in departure frequency is ± 20%.

**Figure 5:**
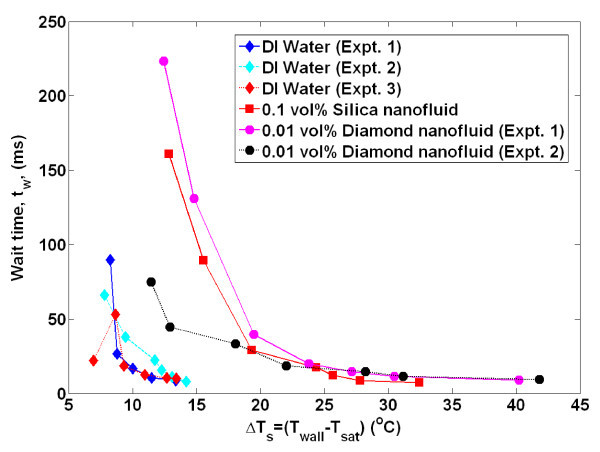
**Average wait time as measured by infrared thermometry**. Uncertainty in wait time is ± 20%.

**Figure 6:**
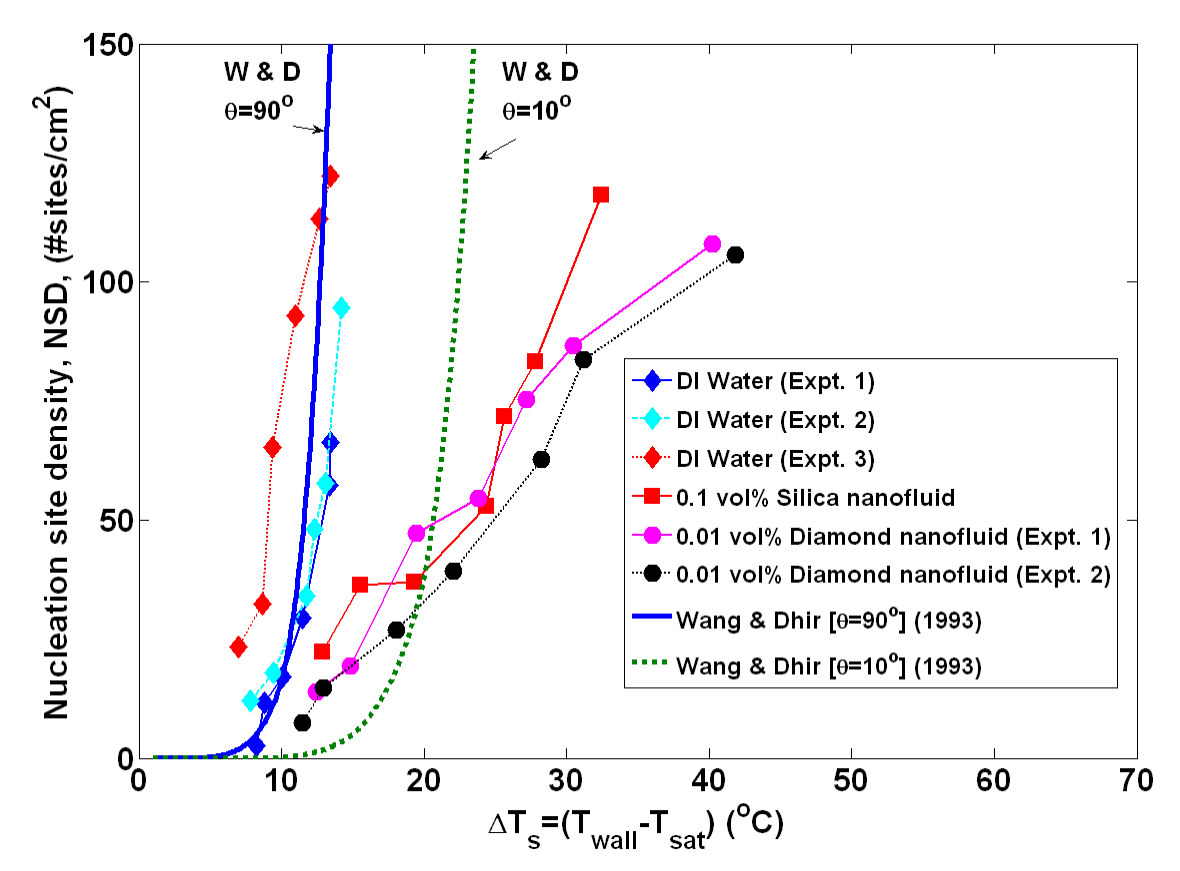
**Active nucleation site density as measured by infrared thermometry**. Uncertainty in nucleation site density is ≤2%.

**Figure 7:**
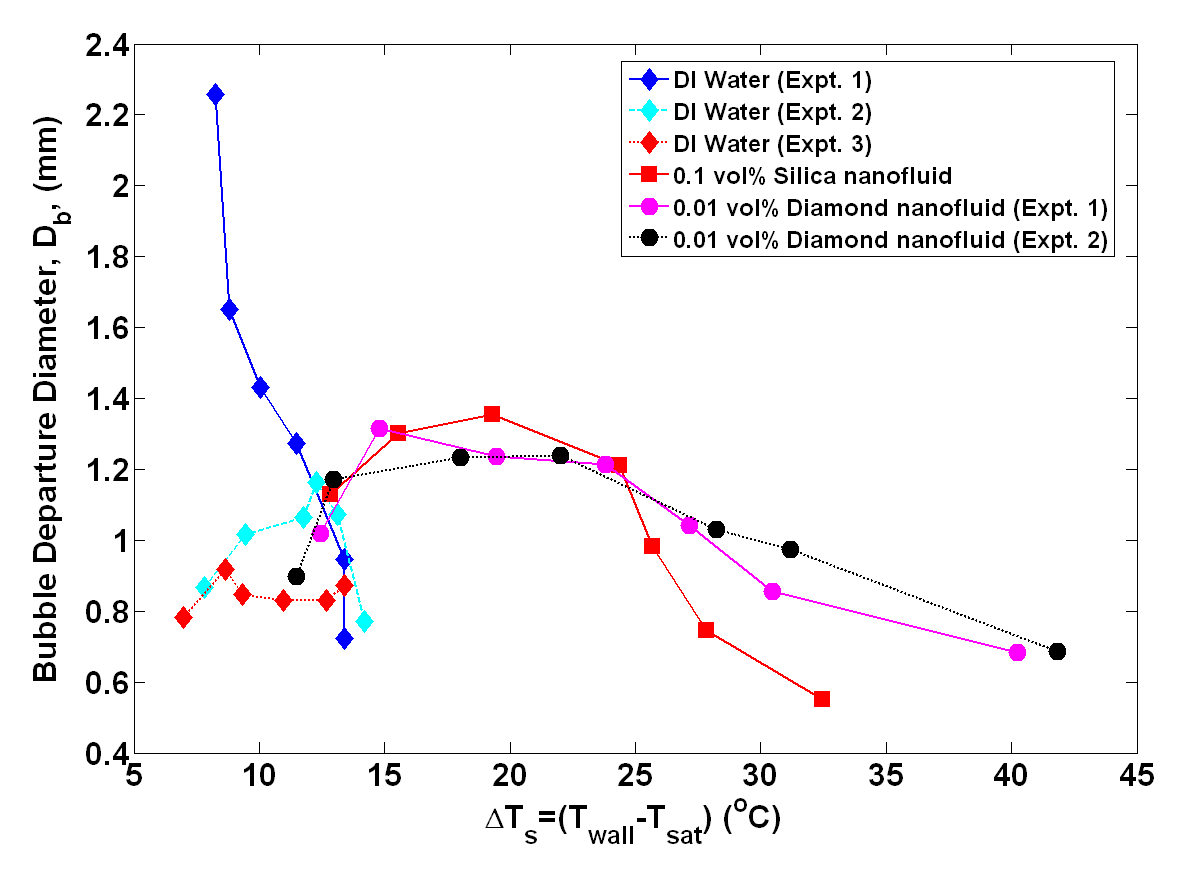
**Average bubble departure diameter as measured by infrared thermometry**. Uncertainty in departure diameter is ± 2%.

**Figure 8:**
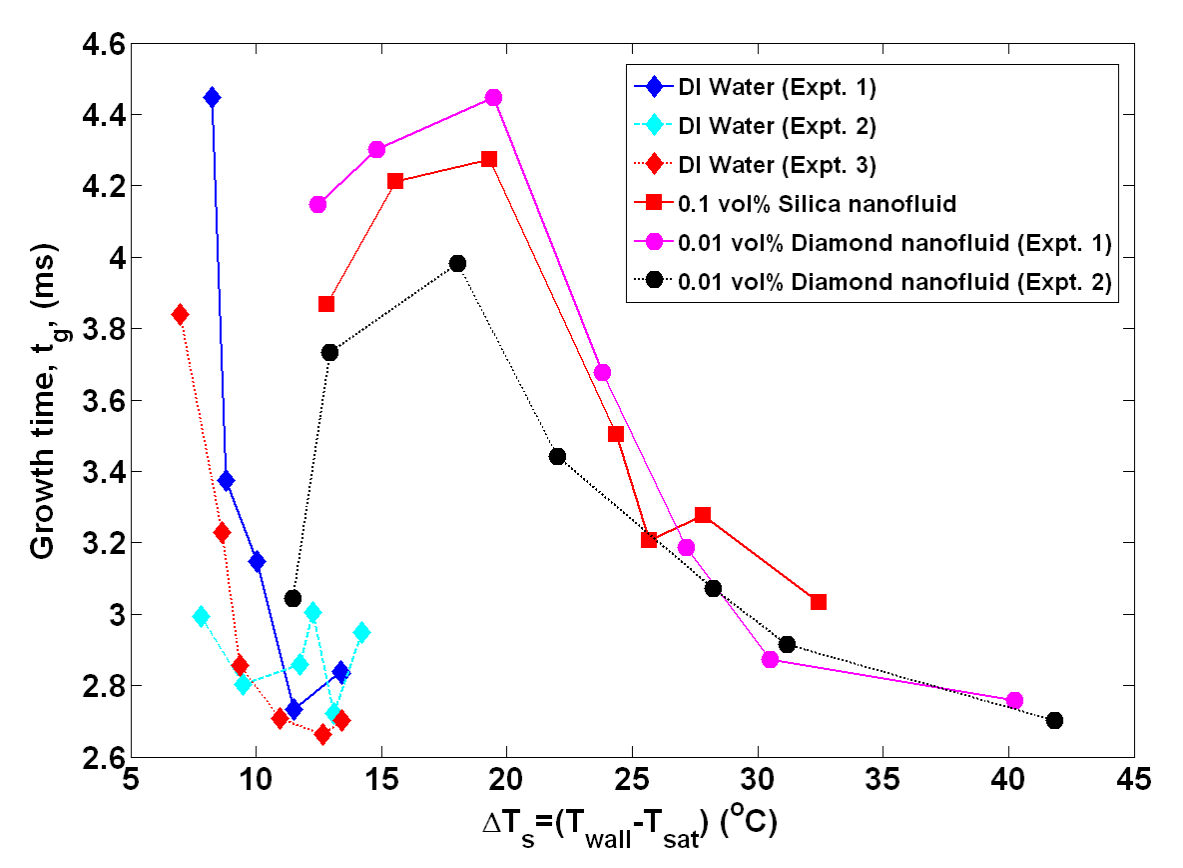
**Average growth time as measured by infrared thermometry**. Uncertainty in growth time is ± 20%.

By obtaining time- and space-resolved temperature data during bubble nucleation, the bubble departure diameter and frequency, growth and wait times, and nucleation site density were directly measured using the techniques detailed in Gerardi et al. [22] and Gerardi [23]. The bubble parameters for each individual nucleation event were tallied. Since boiling is essentially a random phenomenon, for each nucleation site and between nucleation sites, there was a distribution of the parameters; however, we observed that the parameters tend to be distributed narrowly about their mean for a given nucleation site (greater detail is given in the "Appendix"). Therefore, for comparative purposes, only the mean values of the parameters for all nucleation sites are shown in Figures 4,5,6,7,8. It can be seen that for a given wall superheat, the nanofluids have significantly lower bubble departure frequency, higher wait time, and lower nucleation site density with respect to pure water. The implications of these findings will be discussed in "*Data interpretation*."

**Figure 9:**
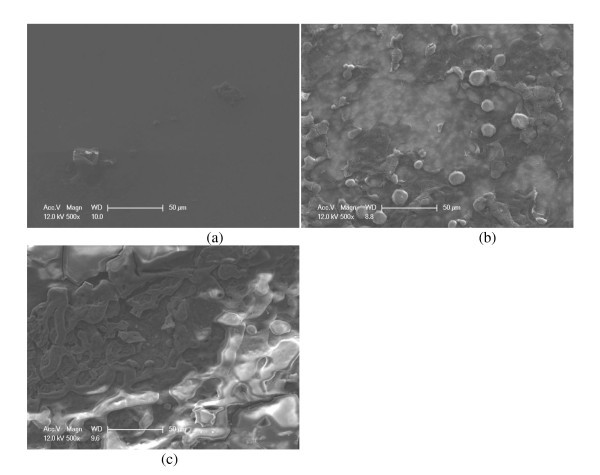
**SEM images (× 500) of ITO heater surface**. After boiling in (**a**) DI water, (**b**) 0.01 vol.% diamond nanofluids, and (**c**) 0.1 vol.% silica nanofluids.

SEM analysis of the heater surface during post-experimental analysis revealed that the surface was clean during pure water boiling (Figure 9a), but a porous layer built up during nanofluid boiling (Figure 9b,c). Energy-dispersive spectrometer analysis of the layer confirmed that it was made of the nanoparticle material. The presence of a porous nanoparticle layer due to particle deposition during nucleate boiling is now well known [18,21]. This particle layer was attached to the substrate well enough to not flake off during handling or when rinsed with a gentle water spray; however, the layer could be removed with moderate abrasion. Confocal microscopy confirmed that the surface roughness (SRa) and surface index (ratio of actual surface area due to peaks and valleys to the projected area viewed) were higher for nanofluid-boiled surfaces than for pure water-boiled surfaces. The measured surface roughness of the water-boiled heater (SRa = 132 nm) was slightly higher than the as-received heater (SRa = 30 nm), while it was significantly higher for the nanofluid-boiled surfaces (900 to 2,100 nm). The surface index for water-boiled surfaces was approximately 1.0 and for nanofluid-boiled surfaces ranged from 1.1 to 1.7. These values were smaller than expected given all of the peaks and valleys created by the nanoparticle deposits, but are consistent with other nanofluid results [26,30].

The porous nanoparticle layer increases surface wettability, which directly affects the boiling phenomena, as will be discussed later. The static contact angle of the as-received heater was approximately 100°, the contact angle of the heaters that were boiled in deionized water [DI] water ranged from 80° to 90°, while the contact angle of the heaters boiled in nanofluids were significantly lower (6° to 16°). There is a slight, but statistically significant, trend of the heaters boiled in silica nanofluids having a lower contact angle than those boiled in diamond nanofluids.

## Data interpretation

As presented above, the nucleate boiling heat transfer coefficient and critical heat flux were found to decrease and increase, respectively, in nanofluids. These behaviors are compatible and related to the surface modification that was observed due to the porous nanoparticle layer deposited via boiling.

### Nucleate boiling heat transfer coefficient deterioration in nanofluids

#### Influence of thermal resistance of nanoparticle surface deposit on boiling curves

The infrared camera measures temperatures on the backside of the ITO heating element. The nanoparticles that deposit onto the surface during nanofluid boiling create a thermal resistance, which tends to shift the boiling curve to the right; therefore, it is examined here in some detail. It is possible to estimate the effective thermal conductivity, *k*_eff_, of the layer using Maxwell's [31] effective medium theory as a function of the thermal conductivities of the particle material, *k*_s_, and the pore-filling fluid, *k*_f_, as:(2)

where(3)

and the porosity, *ε*, is determined with the particles being the solid phase and the pore-filling fluid as the dispersed phase. The interfacial thermal resistance between the nanoparticle material and the pore-filling fluid is included in the effective particle thermal conductivity, *k*_p_, as *k*_p _= *k*_s _+ *αk*_f_, with *α *= *R*_b_*k*_s_/*d*, and *d *is the nanoparticle diameter, as discussed in "*Nanofluid preparation and characterization*." A conservative value for the interfacial thermal resistance has been suggested by Eapen et al. [32] as *R*_b _= 2.5 × 10^-8 ^km^2^/W. Using the maximum porosity for close-packed spherical pores, *ε *= 0.74, and nanoparticle layer thickness of 10 μm (which was shown to be the approximate layer thickness using confocal microscopy), at a heat flux, *q*" = 500 kW/m^2^, assuming steam in the pores (*k*_s _= 0.025 W/mK), the temperature rise on the ITO IR emitting surface would be 0.01°C and 3.1°C for silica and diamond nanoparticle materials, respectively. Since the observed shift in the boiling curve at this heat flux is >15°C, the thermal resistance cannot be the only explanation, even when this analysis has chosen fairly conservative values for porosity. It should be noted that steam was used in this analysis rather than liquid water, which yields a conservative value for the temperature rise since the thermal conductivity of steam is significantly lower than that of water; thus, steam has greater thermal resistance. However, a better understanding of the porosity and fluid that fills the pores is required to make a definitive statement on this subject.

#### Nucleate boiling heat transfer models

The individual bubble parameters jointly determine the macroscopic heat transfer behavior of the surface. To study this behavior, the bubble parameters (*D*_b_, *N*_SD_, *f*_b_, *t*_g_, *t*_w_), whose ensemble-averaged values are shown in Figures 4,5,6,7,8 were used in the popular heat flux partitioning model by Kurul and Podowski [33], which has also been labeled as the 'RPI model' after the authors' university.

The model is based on Bowring's [34] scheme of accounting for the various boiling heat transfer mechanisms separately. Both were primarily developed for flow boiling, but have been extended and applied to pool boiling here.

The heat removed by the boiling fluid is assumed to be through the following contributions:

1. The latent heat of evaporation to form the bubbles (*q*"_e_)

2. Heat expended in the re-formation of the thermal boundary layer following bubble departure, or the so-called quenching heat flux (*q*"_q_)

3. Heat transferred to the liquid phase outside the zone of influence of the bubbles by convection (*q*"_c_)

The total partitioned boiling heat flux is obtained through the addition of the three fluxes as:(4)

Each of the partitioned heat fluxes were expressed to account for the contributions of all of the nucleation sites at a given heat flux and were first described in Gerardi et al. [22]. The expression for the total quenching partitioned heat flux is written as:(5)

with *N*_T _being the total number of nucleation sites at a given heat flux, *n *corresponding to each individual nucleation site, and *A *being the heater area. This expression is reproduced from Gerardi et al. [22] in order to reinforce the concept that the contribution of each nucleation site to the partitioned heat fluxes is accounted for. Expressions for the latent heat of evaporation and convection partitioned heat fluxes are similar and can be found in Gerardi et al. [22].

**Figure 10:**
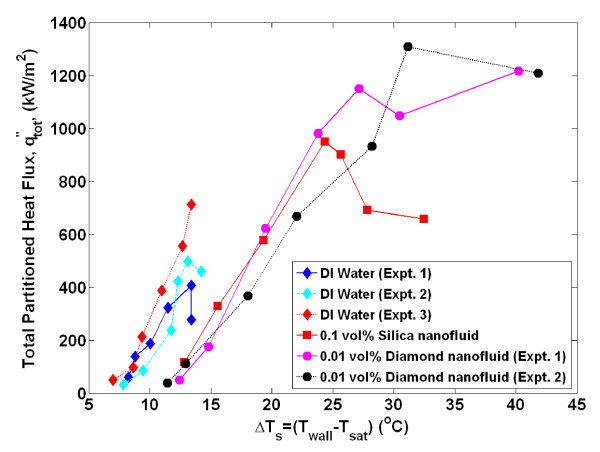
**Total partitioned heat flux predicted by the RPI model**.

A comparison of the nanofluids and water total partitioned boiling heat fluxes is presented in Figure 10. These curves represent the predicted boiling curves for each test using *only *the measured bubble parameters to calculate the heat flux at a given wall superheat. A clear deterioration of the nucleate boiling heat transfer coefficient in nanofluids is seen in agreement with the experimental boiling curve. The dominant heat flux found in the RPI model, the partitioned quench heat flux, *q*"_q_, goes as:(6)

A significant reduction in bubble departure frequency and nucleation site density was found in nanofluids boiling (see Figure 4, Figure 6), which directly correlate to a significant reduction in the heat transfer coefficient predicted by the RPI model. In the next section, the reduction of these bubble parameters is shown to be a result of the surface modification, in particular the increased surface wettability, found for the nanofluid-boiled surfaces.

It should be noted that there is a reduction at high superheat in the case of some total partitioned heat fluxes shown in Figure 10. The exact reason for this is unknown, but it is hypothesized that since the individual partitioned heat fluxes are computed by summing the contribution of all the nucleation sites' bubble parameters, the total heat flux is highly sensitive to small changes in the bubble parameters. In the case of a few experiments, the bubble departure diameter decreased significantly near CHF, which resulted in a reduced calculated partitioned heat flux. Additional experimental data for a wide range of test conditions and nanofluids would be useful for understanding this issue.

#### Surface property influences on bubble parameters

The microcavity theory of bubble growth holds that the required superheat (Δ*T*_sat_) for bubble nucleation is dependent on the cavity size and the contact angle for fixed fluid properties. It is straightforward to show [23] that for a given set of fluid properties, the relationship between the contact angle and wall superheat goes as(7)

where(8)

In the limit of a perfectly wetting system, i.e., *θ *= 0°, the superheat required would be the same as for homogeneous nucleation since *ϕ *= 1, while for an extreme non-wetting system, i.e., *θ *= 180°, no superheat is required for spontaneous bubble growth from a microcavity since *ϕ *= 0. This relationship makes it possible to estimate the difference in superheat required for surfaces with two different contact angles assuming all other properties the same.

The sharp reduction in contact angle of nanofluid-boiled surfaces supports the deterioration of the boiling curve, or shift to the right, that was found for nanofluids. The contact angle for nanofluid-boiled surfaces was approximately *θ *≈ 10°, where *ϕ *≈ 1, which gives no reduction in the required superheat, while the approximate contact angle of water-boiled heaters was *θ *≈ 90°, which results in a value of *ϕ *= 1/2 and a reduction in the required superheat of 1/√2. Thus, the superheat required in water to achieve a given energy of formation is significantly ~1/√2 or 0.707 lower than that for nanofluids. The boiling curve for water is shifted by 27°C to 32°C compared with that of nanofluids at a heat flux of 1,000 kW/m^2^, or approximately a factor of 0.44 to 0.52. Thus, the change in contact angle can explain a significant portion of the deterioration of heat transfer coefficient in nanofluids. Note that this analysis is very approximate since the maximum superheats for the highly wetting nanofluid surfaces are under 50°C, while the prediction for homogenous nucleation of water at atmospheric pressure is approximately 220°C.

It was surprising that for a given wall superheat, the nucleation site density for the nanofluids was lower than that of water (Figure 6), given the formation of the nanoparticle-made porous layer on the boiling surface which likely increases the number of available microcavities for nucleation. However, the observed trend can also be explained by the increased wettability of the nanofluid-boiled surfaces, as discussed next. Carey [35] reported that the active nucleation site density is related to the minimum interface radius during embryo growth, which, in turn, is dependent on the surface contact angle. Wang and Dhir [36] experimentally determined the relationship between contact angle and nucleation site density:(9)

where *N*_c _is the number of microcavities per unit surface area, which Wang and Dhir determined empirically. Wang and Dhir's predictions for the nucleation site density for contact angles of *θ *= 10° and 90°, corresponding to water-boiled and nanofluid-boiled surfaces, respectively, are superimposed over the present experimental data in Figure 6. Wang and Dhir's model predicts a significant decrease in nucleation site density with a reduction in contact angle, consistent with experimental observations. It must be concluded that in our tests, the effect of wettability reduction more than offsets the increase in the number of microcavities, which presumably is brought about by the porous layer.

Additionally, if a greater superheat is required for bubble nucleation in nanofluids, then the wait time (or time it takes for transient conduction to heat the superheated boundary layer to the required superheat) would be expected to be higher than that of water, as was observed. Since the wait time comprises a significant portion (50% to 98%) of the ebullition cycle, it follows that the bubble departure frequency of nanofluids would be lower (*f*_b _= 1/(*t*_w _+ *t*_g_)) than water at a given superheat, as was observed. The additional time it takes to heat the boundary layer of nanofluids to the required superheat can be estimated using a semi-infinite solid analysis assuming a constant heat flux. The boundary layer is idealized to re-form instantly on the heater surface and be heated through one-dimensional conduction with no additional convective effects. The thickness of the thermal boundary layer is assumed to be approximately 200 μm for both water and nanofluids, based on analysis in Gerardi [23]. From Figure 2, for a wall superheat of 14°C, the wall heat flux was approximately 900 and 100 kW/m^2 ^for water and nanofluids, respectively. The time it takes for the entire boundary layer to reach the corresponding superheat is found to be 61 and 280 ms for water and nanofluids, respectively. While these absolute values do not match the experimental wait time data shown in Figure 5, an order of magnitude increase in wait time for nanofluids at a given superheat was observed.

Thus, the increased surface wettability found for the nanofluid-boiled surfaces seems to be the root cause of the deteriorated nucleate boiling heat transfer coefficient in our experiments.

### Critical heat flux increase in nanofluids

#### Effect of wettability on CHF

The hydrodynamic instability theory developed by Zuber [37] suggests that CHF is dependent only on fluid properties. Since nanofluids at the low concentrations used in this study have fluid properties nearly identical to pure water, the hydrodynamic instability theory would predict that nanofluids and water have the same value for CHF, which is contrary to experimental evidence. It is interesting to note that recently, the reliability of the hydrodynamic instability theory has been questioned even for pure fluids (e.g. [29]) based on experimental evidence that micro-hydrodynamics at the heater surface represents the key physics of the burnout process. Three other theories take into account surface wettability on CHF: the macrolayer dryout theory [38,39], hot/dry spot theory [17,40,41], and the bubble interaction theory [42-44]. A thorough review of these theories is presented by Kim et al. [16], where they showed how the hot/dry spot theory of Kandlikar [40] supports an increase in CHF due to the increased surface wettability of the nanofluid-boiled surfaces. Gerardi [23] used the macrolayer dryout theory of Sadasivan et al. [39] and the bubble interaction theory of Kolev [44] to additionally link increased surface wettability with CHF increase.

A discussion of the hot/dry spot theory CHF theory of Kandlikar [40] incorporating measured bubble parameter data to support the influence of the contact angle on CHF was chosen here to convey how the measured bubble parameter data can be used to probe the physical mechanisms in nucleate boiling.

**Figure 11:**
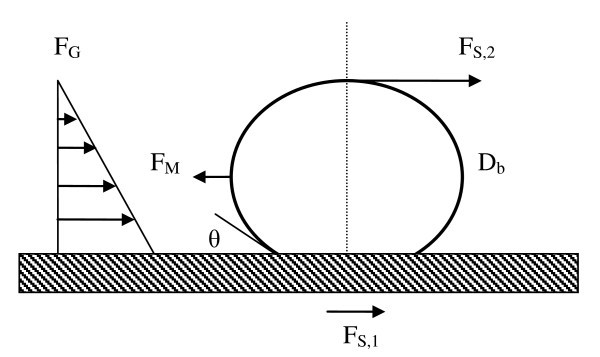
**Forces due to surface tension, gravity, and momentum acting on bubble parallel to the surface**. Adapted from Kandlikar [40].

Kandlikar [40] considered the force balance on the left half of a single bubble at the moment where the force due to change in momentum from evaporation (or evaporation recoil force), *F*_M_, is higher than the sum of the hydrostatic pressure (*F*_G_) and surface tension forces (*F*_S,1 _and *F*_S,2_) holding the bubble in its spherical shape (see Figure 11). This causes the liquid/vapor interface to move rapidly outward along the heater surface, resulting in CHF. Kandlikar assumes that CHF occurs when the force due to the momentum change, *F*_M_, pulling the bubble interface away from the bubble center exceeds the sum of the forces holding the bubble intact, *F*_S,1_, *F*_S,2_, and *F*_G_. The force balance at this moment is:(10)

**Figure 12:**
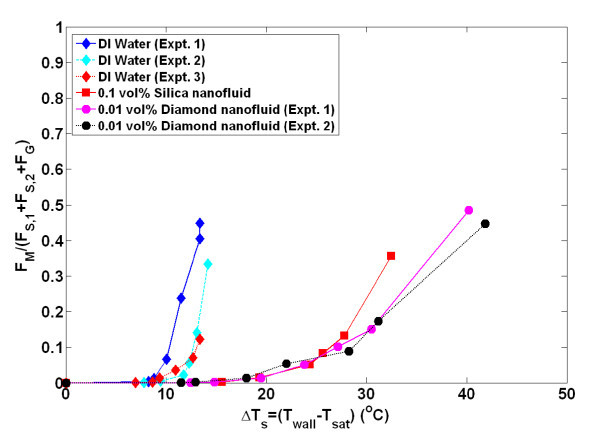
**Ratio of F_M _and (F_S,1 _+ F_S,2 _+ F_G_) vs. wall superheat**. The average bubble diameter, *D*_b_, at a given superheat is used as input along with the contact angle and heat flux.

The present analysis obtained discrete data for the bubble diameter at all wall superheats. The surface contact angle is also known; thus, it is possible to calculate these bubble forces at a given superheat without relying on empirical models or correlations. The average bubble diameter at a given superheat is used for this analysis. The ratio of the force due to the momentum change over the sum of the gravity and surface tension forces is plotted for all superheats in Figure 12.

While none of the experiments reach a value of unity, which is the condition predicted by Kandlikar for CHF, it is remarkable how all cases show the same trend. The value of the force ratio is between 0.33 and 0.50 at CHF for all cases. The fact that a value of unity is never reached is not entirely surprising since there are a number of assumptions in Kandlikar's model, including the bubble shape, area of bubble influence, and the average diameter. However, there is a very clear shift to the right for the nanofluid data, illustrating the reduction in the momentum force with decreasing contact angle. This analysis clearly demonstrates the effect of contact angle on the forces theorized to dominate at CHF. It also is the first time actual experimental data on bubble parameters have been used to quantify these forces and relate them to the CHF condition.

Kandlikar uses the force balance at CHF to solve for the heat flux where CHF is reached, *q*"_CHF_:(11)

where(12)

**Figure 13:**
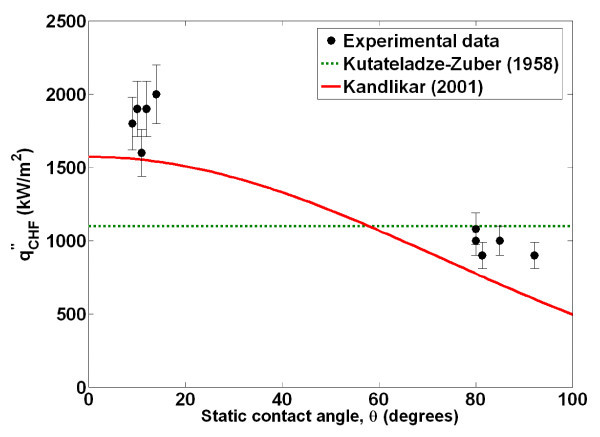
**Effect of contact angle on critical heat flux**.

A comparison of Kandlikar's predicted CHF values with the experimental data is shown in Figure 13, with good qualitative agreement between the two. Thus, we have experimentally confirmed that the hot/dry spot theory of Kandlikar supports an increase in CHF due to an increase in surface wettability through direct measurement of bubble parameters. The CHF result is consistent with that reported in Truong et al. [24] for alumina, zinc oxide, and diamond nanofluids.

#### Effect of other surface changes on CHF

In addition to increasing the surface wettability, the nanoparticle layer deposited on the surface alters the thermal properties of the surface. The particle layer may promote radial heat dissipation of a local hot spot via conduction, alter liquid replenishment to the surface through capillary wicking through the thin porous layer, or increase surface-to-fluid heat transfer through fin action. Each of these possibilities was considered by Gerardi [23] for the surfaces and conditions used in these experiments. The radial heat dissipation and fin effect were ruled out as major contributors to CHF enhancement. The porous effect was not studied in detail since porosity was not directly measured in this study. However, Kim and Kim [21] explored the effect of porous nanoparticle layers on CHF due to capillary wicking and showed that a portion of the CHF increase could be explained by capillary wicking.

## Conclusions

Infrared thermometry was used to obtain time- and space-resolved information on nanofluid pool boiling phenomena. This approach provides a detailed method for investigating the fundamentals of nucleate boiling. Data on bubble departure diameter and frequency, growth and wait times, and nucleation site density were measured for all nucleation sites on the heater surface. The experimentally determined decrease in nucleate boiling heat transfer and increase in critical heat flux were examined in detail with this method. While the conditions tested in this work were limited (particularly in nanofluid types and particle volume fraction range), this study represents a significant first step toward a complete understanding of boiling heat transfer in nanofluids. The IR thermometry approach was shown to be capable of providing new insight into nanofluid boiling phenomena. The main findings of the study relevant to the specific nanofluids studied are as follows:

-The nanoparticle layer increases the heater surface wettability which was shown to be responsible for the observed increase in wait time between bubble nucleation events (thus lower departure frequency) and lower nucleation site density.

-The RPI heat flux partitioning model, directly informed by our bubble parameter experimental data, suggests that the decrease in bubble departure frequency and nucleation site density are responsible for the observed deterioration in the nucleate boiling heat transfer coefficient for nanofluids.

-Kandlikar's hot/dry spot theory for CHF, to which our data on nucleation site density, bubble departure diameter, and frequency were directly fed, suggests that the reduction in contact angle sharply reduces the momentum force acting on a bubble, for a given wall superheat, which delays CHF.

## Abbreviations

**Nomenclature ***A*: area of heater (m^2^); CHF: critical heat flux (kW/m^2^); *d*: nanoparticle diameter (nm); *D*_b_: bubble departure diameter (m); *F*: bubble force per unit length (N/m); *f*_b_: bubble departure frequency (Hz); *g*: gravity acceleration (m/s^2^); *h*: heat transfer coefficient (W/m^2^-K); *h*_fg_: latent heat of evaporation (J/kg); *K*: constant (dimensionless); *k*: thermal conductivity (W/m-K); *N*_c_: number microcavities per unit surface area (#sites/cm^2^); *N*_SD_: nucleation site density (#sites/cm^2^); *N*_T_: total number of nucleation sites (#); *q*": heat flux (W/m^2^); *R*_b_: interfacial thermal resistance (Km^2^/W); SRa: average surface roughness (μm); *T*: temperature (°C); *t*: time (s); Δ*T*_sat_: surface superheat (°C); **Greek ***α*: effect of interfacial resistance; *α*: thermal diffusivity (m^2^/s); *ε*: porosity; *θ*: contact angle (degrees); *ρ*: density (kg/m^3^); *σ*: surface tension (N/m); *ϕ*: angle of inclination (degrees); *ϕ*: contact angle factor; **Subscript **c: convection partitioned heat flux; e: evaporation partitioned heat flux; eff: effective; f: pore filling fluid; g: growth; G: hydrostatic pressure; l: liquid; M: momentum force due to evaporation; p: effective particle thermal conductivity; q: re-formation of thermal boundary layer partitioned heat flux; s: solid nanoparticle material; S,1: surface tension force (fluid/vapor/solid); S,2: surface tension force (vapor/fluid); sat: saturation; v: vapor; w: wait; w: wall.

## Competing interests

The authors declare that they have no competing interests.

## Authors' contributions

CG conducted the boiling experiments, carried out the analysis, and drafted the manuscript. JB, L-wH, and TM conceived of the study, participated in its design, and scope of analysis. All authors read and approved the final manuscript.

## Appendix

### Bubble parameter data distribution for a single nucleation site

**Figure 14:**
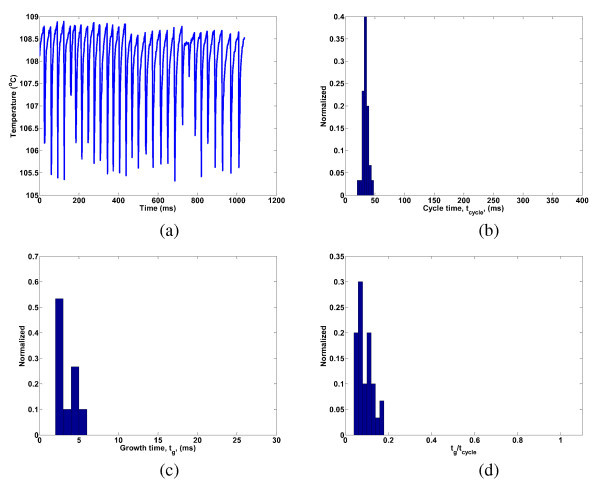
**Bubble cycle time distributions for a single DI water nucleation site (Expt. 2, q"= 50 kW/m**^**2**^**)**. Shown are the (**a**) temperature history and distributions of the (**b**) cycle time, (**c**) growth time, and (**d**) ratio of growth to cycle time.

Data from many bubble cycles at each nucleation site are used to arrive at the average values for the departure frequency, growth time, and wait time that are used in the heat transfer coefficient and CHF models discussed in "*Data interpretation*." There is, of course, some variability in these parameters even for a given nucleation site. In order to provide an example of this variability, a single nucleation site for DI water (Expt. 2, *q*" = 50 kW/m^2^) is chosen. For this nucleation site, a 1.0-s temperature history along with the distribution of the cycle time, *t*_cycle _(1/*f*_b_), growth time, *t*_g_, and growth-to-cycle time ratio are shown in Figure 14. Other nucleation sites, fluids, and heat fluxes have distributions that are correspondingly narrow.

**Figure 15:**
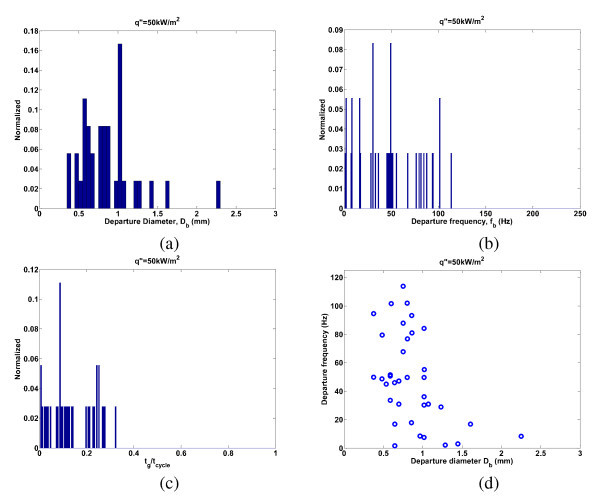
**Bubble parameter distributions for all DI water nucleation sites (Expt. 2, q"= 50 kW/m**^**2**^**)**. Shown are the distribution of (**a**) bubble departure diameter, (**b**) departure frequency, (**c**) ratio of bubble growth time to cycle time, and (**d**) the relationship between frequency and diameter for a given nucleation site.

There also is some variability in the bubble departure diameter, frequency, and growth and wait times between individual nucleation sites at a given heat flux (or superheat). While the individual nucleation site values were used in the analysis of the heat transfer coefficient and CHF models in the present paper, the ensemble averages for these parameters were discussed in "*Experimental results*" and reported in Figures 4,5,7,8 in order to allow quick comparison between the water and nanofluid data. In order to provide an example of the variability of bubble parameters across nucleation sites, the data for DI water (Expt. 2, *q*" = 50 kW/m^2^) is chosen again. For this experiment, the distribution of the bubble departure diameter, departure frequency, and ratio of bubble growth time to the cycle time are shown in Figure 15. Also shown in Figure 15d is the relationship between the frequency and departure diameter for each nucleation site for this experiment. There is a wide distribution in all of these parameters across the nucleation sites, significantly wider than for an individual nucleation site, suggesting that each nucleation site is fairly unique.
